# The Roles of Magnetic Resonance-Guided Focused Ultrasound in Pain Relief in Patients With Bone Metastases: A Systemic Review and Meta-Analysis

**DOI:** 10.3389/fonc.2021.617295

**Published:** 2021-08-11

**Authors:** Xiaying Han, Runzhi Huang, Tong Meng, Huabin Yin, Dianwen Song

**Affiliations:** ^1^Department of Orthopedics, Shanghai General Hospital, Shanghai Jiaotong University School of Medicine, Shanghai, China; ^2^Department of Orthopedics, The First Affiliated Hospital of Zhengzhou University, Zhengzhou, China; ^3^Division of Spine, Department of Orthopedics, Tongji Hospital affiliated to Tongji University School of Medicine, Shanghai, China; ^4^Tongji University School of Medicine, Shanghai, China

**Keywords:** MRgFUS, bone metastases, safety, efficacy, cancer pain

## Abstract

**Objective:**

Cancer pain, the most common skeleton-related event of bone metastases, significantly disturbs patients’ life. MRI-guided focused ultrasound (MRgFUS) is a therapeutic option to relieve pain; however, its efficacy and safety have not been fully explored. Therefore, we aim to conduct a meta-analysis on studies reporting MRgFUS for patients with bone metastases.

**Methods:**

Randomized controlled trials (RCT) and non-RCTs on MRgFUS treatment for patients with bone metastases were collected using PubMed, MEDLINE In-Process (US National Library of Medicine), National Institutes of Health (US National Library of Medicine), Embase (Elsevier), Web of Science, CINAHL, and the Cochrane Library between August 2007 and September 2019. Data on quantitative pain assessment before/after MRgFUS, response rate, and complication were extracted and analyzed.

**Results:**

Fifteen eligible studies with 362 patients were selected in this meta-analysis. The average pain score was 6.74 (95% CI: 6.30–7.18) at baseline, 4.15 (95% CI: 3.31–4.99) at 0–1 week, 3.09 (95% CI: 2.46–3.72) at 1–5 weeks, and 2.28 (95% CI: 1.37–3.19) at 5–14 weeks. Compared with baseline, the pain improvement at 0–1 week was 2.54 (95% CI: 1.92–3.16, *p* < 0.01), at 1–5 weeks was 3.56 (95% CI: 3.11–4.02, *p* < 0.01), and at 5–14 weeks was 4.22 (95% CI: 3.68–4.76, *p* < 0.01). Change from baseline in OMEDD at 2 weeks after treatment was −15.11 (95% CI: −34.73, 4.50), at 1 month after treatment was −10.87 (95% CI: −26.32, 4.58), and at 3 months after treatment was −5.53 (95% CI: −20.44, 9.38). The overall CR rate was 0.36 (95% CI: 0.24–0.48), PR rate was 0.47 (95% CI: 0.36–0.58), and NR rate was 0.23 (95% CI: 0.13–0.34). Among 14 studies including 352 patients, 93 (26.4%) patients with minor complications and 5 (1.42%) patients with major complications were recorded.

**Conclusion:**

This meta-analysis identifies MRgFUS as a reliable therapeutic option to relieve cancer pain for patients with metastatic bone tumors with controllable related complications.

## Introduction

Bone is the third most common distant metastatic organ secondary to lung and liver (1), and about 30% of patients with malignancies have experienced bone metastases during their follow-up (2). Bone metastases often induce skeleton-related events, such as local pain, pathologic fracture, and spinal cord compression (3), which subsequently reduce the life quality and decrease the overall survival (OS) (4). Among all the symptoms, cancer pain is the most common one and significantly disturbs patients’ normal life. Thus, there is a pressing need to control it effectively and improve the quality of life.

Generally, conventional radiotherapy (RT) is the main therapeutic option to relieve local pain and restore normal function in patients with symptomatic bone metastases. In terms of pain relief, RT provides a relief rate of 60% to 80% (5, 6). Besides, analgesics are also optional therapeutic methods and achieve good pain control. However, their adverse effects, such as drug resistance and addiction, cannot be neglected (7, 8). With the advance of medical technology, thermal ablation is regarded as an alternative local therapy for painful bone metastasis with excellent response rates and safety (9–13). It can directly induce irreversible damage or coagulative necrosis of tumor cells by heat effects (14). Generally, thermal ablation includes radiofrequency ablation (RFA), microwave ablation (MWA), cryoablation (CA), laser ablation (LA), and magnetic resonance-guided focused ultrasound (MRgFUS) ablation (15).

Focused ultrasound is a non-invasive technology proposed by Lele et al. 40 years ago (16). It delivers acoustic energy to heat the lesion in an ablation temperature (over 65°C) locally and subsequently induces local tumor tissue coagulation and necrosis. In addition, it also destroys the nerve on the affected periosteum, which alleviates cancer pain in both osteoblasts and osteolytic bone metastases (17–19). MRI-guided focused ultrasound (MRgFUS) therapy is a novel focused ultrasound method, which enables oncologists to perform ablation precisely and provides real-time temperature monitoring by MR thermometers (20, 21). Compared with other invasive and interventional therapies with non-uniform dose distribution, the distribution of MRgFUS therapeutic dose is uniform (22).

Although preliminary clinical studies of MRgFUS have shown excellent response rates and safety to relieve painful bone metastases, reliable data regarding long-term efficacy and complications are still scarce. In this regard, we aim to perform a meta-analysis to evaluate the pain relief efficacy and safety of MRgFUS in patients with bone metastases. Our results may provide a more reliable basis for the clinical applications of MRgFUS in painful bone metastases.

## Materials and Methods

The study was in accordance with the guidelines included in the Preferred Reporting Items for Systematic Reviews and Meta-Analyses (PRISMA) Statement (23) and Cochrane’s guidelines for systematic reviews of interventions (15, 24). The PubMed, MEDLINE In-Process (US National Library of Medicine), National Institutes of Health (US National Library of Medicine), Embase (Elsevier), Web of Science, CINAHL, and the Cochrane Library were chosen to search literatures for original clinical studies regarding the roles of MRgFUS in pain relief in patients with bone metastases. Keywords included “focused ultrasound,” “MRgFUS,” “HIFU,” “painful,” “bone,” “bone metastases,” “pain management,” and their expansions (15).

The inclusion criteria were as follows: the type of study included randomized controlled trial (RCT), non-RCT between August 2007 and September 2019, and the research objects were patients with bone metastases. The intervention measures were that the trial group was given MRgFUS with cases more than 10; it was not limited whether there was a control group, and if a control group was set, the intervention measures were not limited. The exclusion criteria were as follows: studies reporting molecular, focused, *in vitro*, or animal studies, or patients who also underwent other therapies (e.g., RT, cementoplasty) (15); the evaluation indicators do not include pain grade scores; case report; and review. We used the “Methodological index for non-randomized studies” (MINORS) items to assess the quality of the included single-arm clinical research methodology (25).

The main evaluation indicators include pain grade scores, the change from baseline in oral morphine equivalent dose (OMEDD), and the response rate. All but one paper included in the study used 10-point scales to assess pain and the one paper used 100-point scales. The data of the one paper was transformed from a 100-point scale into a 10-point scale for comparison purposes (15). Change from baseline in OMEDD means changes from baseline in the OMEDD at each evaluation point after treatment of patients with bone metastases. Pain grade scores were evaluated at four time intervals: baseline (pretreatment), 0–1 week, 1–5 weeks, and 5–14 weeks. If an author reports multiple pain assessments in the same time interval (e.g., 6 and 7 weeks), only the latest one is considered (e.g., a 7-week evaluation reported in a time interval of 5 to 14 weeks) (15). The change of OMEDD was recorded at three evaluation points after treatment of patients with bone metastases: at 2 weeks, at 1 month, and at 3 months. The response rate includes complete response (CR), partial response (PR), and no response (NR). CR is defined as a pain score of 0 without medication increase; PR is defined as a drop of 2 points on a 10-point scale without an increase in pain medications or a drop of 25% in pain medication without increase in the reported pain score; NR is defined as no drop of score and no changes in medication use (26, 27). Other evaluation indicators include QLQ-BM22 subscale scores, QLQ-C15, QLQ-C30, biomarker evaluation including alkaline phosphatase and lactic acid dehydrogenase, and median overall survival time. Secondary evaluation indicators include the types of complications and the complication ratio with respect to major complications and minor complications. Complications were evaluated and classified based on the unified and standardized grading system developed by the Society of Interventional Radiology (SIR) (15, 28, 29).

Two independent investigators (X.H. and R.H.) extracted the following data from each included study: the first author, year of publication, sample size, type of study, follow-up time, and evaluation indicators. Any discrepancy was resolved through discussion. All data obtained was carefully checked to ensure accuracy (30). The literature screening and data extraction were independently completed and cross-checked by two researchers. If there is a disagreement, it will be decided by the third researcher (T.M.).

The STATA statistical software (Version 8, STATA, College Station, TX) was used for meta-analysis of the pain grade scores in each follow-up period. The meta-analysis was performed using the generic inverse variance. *I* statistic was used to access statistical heterogeneity among studies. *I* values of 25%, 50%, and 75% defined mild, moderate, and severe heterogeneity, respectively (30). The fixed-effects model was used to conduct the meta-analysis of non-heterogeneity research; the random-effects model was used to conduct the meta-analysis of heterogeneous research (31). The presence of publication bias was accessed by using the funnel plot. *p* < 0.05 meant statistically significant (30).

## Results

**Figure 1:**
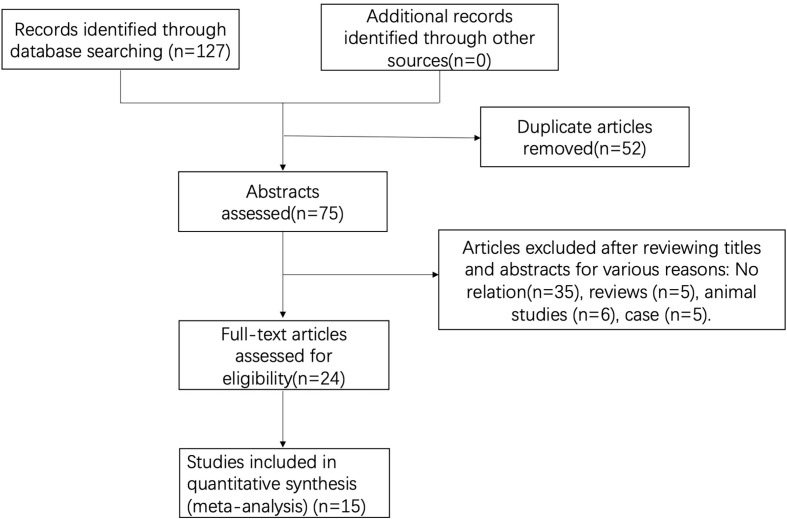
Flow diagram of the literature search and study selection process.

A total of 127 studies were initially screened, and 52 duplicate studies were removed. After reviewing available titles and abstracts, 60 studies were excluded for various reasons: no relation (*n* = 35) or other types of studies (i.e., review, *n* = 5; case report, *n* = 5). Finally, a total of 15 studies were included in this study (**Figure 1**).

**Figure 2:**
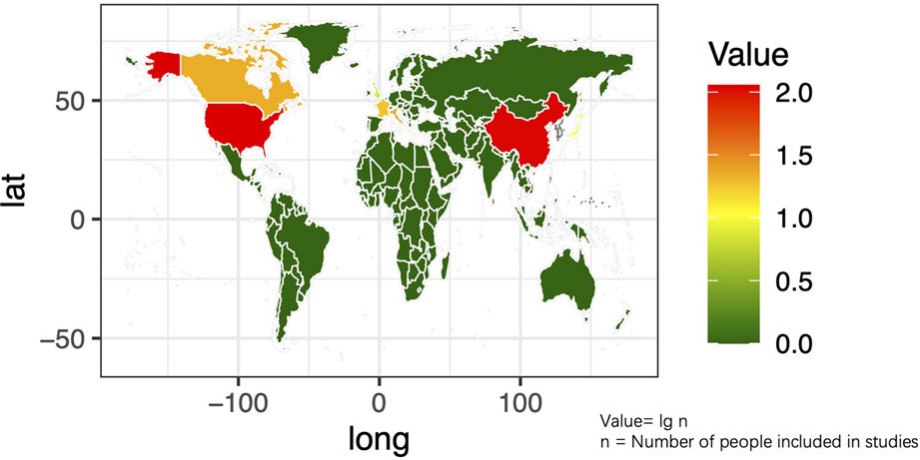
The distribution of the research population in the included studies on the world map.

**Figure 3:**
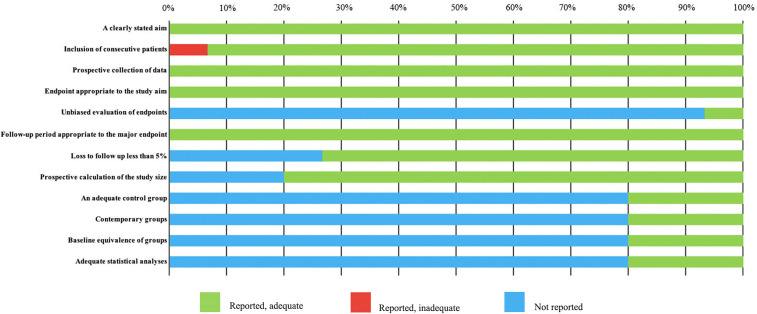
MINORS quality assessment.

The basic characteristics of the selected studies are presented in **Table 1** including the patients’ characteristics and treatment parameters. In total, this study comprised 362 patients. The research objects in most studies were adults and one study included all children (40). The distribution of the study samples were shown on the world map and the patients were mainly from China (*n* = 112), the United States (*n* = 112), Israel (*n* = 38), Italy (*n* = 23), France (*n* = 17), Netherlands (*n* = 15), Canada (*n* = 21), Japan (*n* = 10), South Korea (*n* = 5), and the United Kingdom (*n* = 9) (**Figure 2**). As most of the included studies were single-arm clinical researches, we chose the MINORS items to evaluate the quality of studies. Each item has a score of 0–2, and the highest score is 24. The quality assessment is shown in **Table 2** and **Figure 3**. The quality of studies conducted by the MINORS score and the mean MINORS score was 14.6 (range: 9–24). Most of the studies scored low on unbiased assessment of outcomes due to lack of blinding and control groups. The funnel plot was used to detect bias in studies included in the meta-analysis and no publication bias was found (**Supplementary Figure 1**).

**Table 1:** Basic characteristics of the included 15 studies.

Author-publication time	*n*	Age of patient, range, (mean)	Metastasis type	Metastasis location, *n* (%)	Primary tumor, *n* (%)	Type of Study	MRgFUS treatment length; range, (mean)	Sonications, range, (mean)	Sonication energy, range, (mean)	Acoustic power (mean)	Follow-up time	Evaluation index
Anzidei-2016 (27)	23	37–82 (63.6)	Osteolytic 10 (43.5); Sclerotic 7 (30.4); Mixed 6 (26.1)	Non-axial skeleton 11 (47.8); Axial skeleton 12 (52.2)	Lung 6 (26.1); Breast 5 (21.7); Prostate 4 (17.4); Colon 3 (13.1); Others 5 (21.7)	Non-RCT	55–180 min (110.4 min)	15–29(23.5)	860–2899 J (1644 J)	114 W	1, 3, and 6 months after treatment	VAS; CR; PR; NR
Bertrand-2018 (32)	17	46–89 (61.4)	\	Non-axial skeleton 11 (64.7); Axial skeleton 6 (35.3)	Lung 7 (41.1); Breast 5 (29.4); Kidney 2 (11.8); Larynx 1 (5.9); Endometria 1 (5.9); Prostate 1 (5.9);	Non-RCT	\	6–40(15.1)	\	100 W	Before treatment, at 1 week, and at 1 month after treatment	VAS; OMEDD; CR; PR; NR
Catane-2007 (33)	13	\	Osteolytic 7 (53.8); Osteoblastic 5 (38.5); Mixed 1 (7.7)	Non-axial skeleton 11 (84.5); Axial skeleton 2 (15.4)	Breast 4 (30.8); Prostate 2 (15.4); Bile duct (7.8); Ovaries 1 (7.8); Esophagus1 (7.8); Kidney 1 (7.8); Rectum 1 (7.8); Undetermined 1 (7.8)	Non-RCT	22–162 min (71.8min)	11–39 (25)	418–1890 J (1067.6 J)	54 W	Mean follow-up was 59 days: 0, 3, 15, 30, 90, 180 days after treatment	VAS
Chan-2017 (34)	10	42–78 (64.5)	\	Non-axial skeleton 9 (90); Axial skeleton 1 (10)	Breast 2 (20); Prostate 2 (20); Neuroendocrine NOS 1 (10); Liver 1 (10); Esophagus 1 (10); Pancreas 1 (10); Lung 1 (10); Orbit 1 (10)	Non-RCT	42–78 min (64.5min)	12–51 (29.8)	300–1400J (850 J)	34.1 W	Before, day 14, and day 30 after treatment	Brief Pain Inventory scores; CR; PR; NR
Chen-2018 (19)	26	54.7	Osteolytic 9 (34.6); Osteoblastic 11 (42.3); Mixed 6 (23.1)	Non-axial skeleton 18 (69.2); Axial skeleton 8 (30.8)	Lung 12 (46.2); Breast 5 (19.2); Colon 3 (11.5); Prostate 3 (11.5); Thyroid 2 (7.7); Kidney 1 (3.8)	Non-RCT	\	\	\	\	0, 2, 4, 6, 8, 10, 12 months after treatment	VAS; QLQ-BM22
Huisman-2014 (35)	11	53–86 (62.1)	Osteolytic 6 (55.5); Osteoblastic 1 (9.1); Mixed 4 (36.4)	Non-axial skeleton 6 (55.5); Axial skeleton 5 (45.5)	Kidney 2 (18.2); Colorectal 2 (18.2); Breast 2 (18.2); Sarcoma 1 (9.1); Prostate 1 (9.1); Lung 1 (9.1); Colorectal 1 (9.1); Mesothelioma 1 (9.1)	Non-RCT	20–73 min (458min)	6–28 (15.5)	300–3000 J (1492.3 J)	20–150 W (85.4 W)	Before treatment, 3 days and 1 month after treatment	NRS; CR; PR; NR
Hurwitz-2014 (36)	112	9.1–83.6 (62.7)	Osteoblastic 25 (22.3); Osteolytic 59 (52.7); Mixed 27 (24.1); Unknown 1 (0.9)	Non-axial skeleton 84 (75); Axial skeleton 28 (25)	Breast 34 (30.4) Prostate 15 (13.4) Kidney 9 (8.0) Lung 17 (15.2) Missing 2 (1.8) Other 35 (31.2)	RCT	83 min	\	\	\	3, 7, 14, 30, 60, 90 days and 1 year after treatment	VAS; OMEDD; CR; PR; NR
Li-2010 (22)	12	32–72 (46.8)	\	Non-axial skeleton 5 (41.7); Axial skeleton 7 (58.3)	Lung (4); Liver (5); Kidney (1); Mediastinum (1); Colon (1)	Non-RCT	27.5–647.6 min (230.9 min)	\	\	\	0, 1, 4–6 weeks, 4–6 months after treatment	Biomarker evaluation; CR; PR; NR
Liberman-2008 (37)	25	40–85 (61)	Osteolytic 20 (64.5); Osteoblastic 10 (32.3); Mixed1 (3.2)	Non-axial skeleton 27 (87.1); Axial skeleton 4 (13)	Kidney 6 (19.4); Colorectal 2 (6.5); Lung 1 (3.2); Breast 11 (35.5); Prostate 5 (16.1); Other 6 (19.4)	Non-RCT	22–162 min (66 min)	8–32 (17.3)	440–1890 J (1135 J)	\	0, 3, 13, 30, 90 days after treatment	VAS; OMEDD; CR; PR; NR
Lee-2017 (13)	21	40–83 (59)	\	Non-axial skeleton 20 (95); Axial skeleton 1 (5)	Breast 4 (19); Nasopharyngeal 4 (19); Colorectal 3 (14); Non-small-cell lung 3 (14); Liver (10); Prostate 2 (10); Kidney 1 (5); Cervical 1 (5); Thymic 1 (5).	RCT	\	\	\	\	0, 8, 13, 30, 90 days after treatment	VAS; OMEDD; CR; PR; NR; median overall survival time
Namba-2019 (38)	10	41–80 (69)	Osteolytic 4 (40); Osteoblastic; and Mixed	Non-axial skeleton 8 (80); Axial skeleton 2 (20)	Prostate 2 (20); Myeloma 2 (20); Liver 1 (10); Uterus 1 (10); Lung 1 (10); Thyroid 1 (10); Breast 1 (10); Adenoid cystic carcinoma 1 (10);	Non-RCT	\	\	976 J	\	0, 7, 30, and 90 days after treatment	NRS; NR
Gianfelice-2008 (39)	11	38–84 (58.6)	Osteolytic 8 (72.7); Osteoblastic 2 (18.2); Mixed 1 (9.1)	Non-axial skeleton 11 (100); Axial skeleton 0 (0)	Breast 5 (45.5); Kidney 4 (36.4); Liver 1 (9.1); Lung 1 (9.1)	Non-RCT	28–103 min	12–18	466–1853 J	\	0, 1, 3, 14, 30, 90 days after treatment	VAS; CR; PR; NR
Wang-2019 (40)	30	3–14 (4.27)	Osteoblastic 4 (46.67); Osteolytic 5 (16.67); Mixed type 11 (36.67).	Non-axial skeleton 15 (50); Axial skeleton 15 (50)	Neuroblastoma 12 (40.00); Acute leukemia 7 (23.33); Nephroblastoma 6 (20.00); Lymphoma 5 (16.67); Other 2 (6.67)	Non-RCT	123 ± 21 min	13 ± 8	\	\	0, 7, 30, 60, 90 days after treatment	VAS+NRS
Harding-2018 (41)	18	36–72 (57)	\	Non-axial skeleton 15 (50); Axial skeleton 1 (5.6)	Breast 7 (38.9); Lung 4 (22.2); Liver 4 (22.2); Renal 3 (16.7)	Non-RCT	\	\	\	\	0, 7, 14, 30, 60, and 90 days after treatment	QLQ-C15-PAL; NR; QLQ-BM22
Gu-2015 (42)	23	45–73 (59)	\	\	\	Non-RCT	\	\	\	\	0, 7, 14, 30, 60, and 90 days after treatment	NRS

**Table 2:** MINORS quality assessment.

Author-publication time	A clearly stated aim	Inclusion of consecutive patients	Prospective collection of data	Endpoint appropriate to the study aim	Unbiased evaluation of endpoints	Follow-up period appropriate to the major endpoint	Loss to follow up less than 5%	Prospective calculation of the study size	An adequate control group	Contemporary groups	Baseline equivalence of groups	Adequate statistical analyses	Total
Anzidei-2016 (27)	2	2	2	2	0	2	2	2	0	0	0	0	12
Bertrand-2018 (32)	2	2	2	2	0	2	2	2	0	0	0	0	14
Catane-2007 (33)	2	1	2	2	0	2	0	0	0	0	0	0	9
Chan-2017 (34)	2	2	2	2	0	2	0	0	0	0	0	0	10
Chen-2018 (19)	2	2	2	2	0	2	2	2	0	0	0	0	14
Huisman-2014 (35)	2	2	2	2	0	2	0	2	0	0	0	0	12
Hurwitz-2014 (36)	2	2	2	2	2	2	2	2	2	2	2	2	24
Li-2010 (22)	2	2	2	2	0	2	2	0	0	0	0	0	12
Liberman-2008 (37)	2	2	2	2	0	2	2	2	0	0	0	0	14
Lee-2017 (13)	2	2	2	2	0	2	2	2	2	2	2	2	22
Namba-2019 (38)	2	2	2	2	0	2	0	2	2	2	2	2	20
Gianfelice-2008 (39)	2	2	2	2	0	2	2	2	0	0	0	0	14
Wang-2019 (40)	2	2	2	2	0	2	2	2	0	0	0	0	14
Harding-2018 (41)	2	2	2	2	0	2	2	2	0	0	0	0	14
Gu-2015 (42)	2	2	2	2	0	2	2	2	0	0	0	0	14
Not reported	0	0	0	0	14	0	4	3	12	12	12	12	69
Reported, inadequate	0	1	0	0	0	0	0	0	0	0	0	0	1
Reported, adequate	15	14	15	15	1	15	11	12	3	3	3	3	110

**Figure 4:**
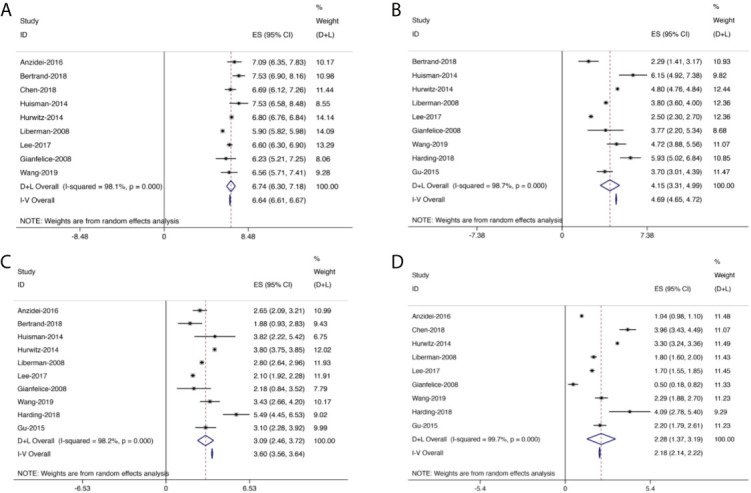
Forest plot for inclusion of all included studies assessing pain at different time points; diamonds represent overall pain scores for random and fixed effect models with 95% CI. **(A)** Pain assessment at baseline. **(B)** Pain assessment at 0–1 week. **(C)** Pain assessment at 1–5 weeks. **(D)** Pain assessment at 5–14 weeks.

**Figure 5:**
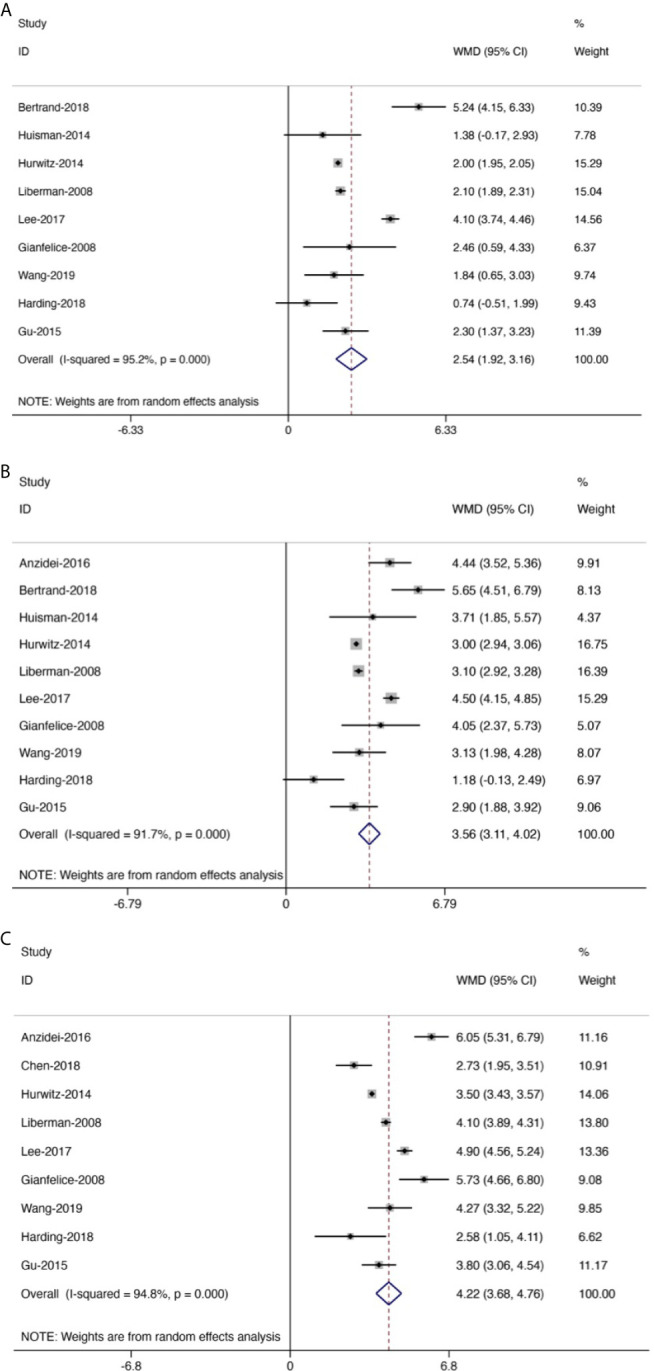
Forest plot for inclusion of all included studies assessing pain improvement at different time points compared with baseline; diamonds represent overall pain scores for random and fixed effect models with 95% CI. Meta-analysis of pain improvement at different time points. **(A)** At 0–1 week. **(B)** At 1–5 weeks. **(C)** At 5–14 weeks.

**Figure 6:**
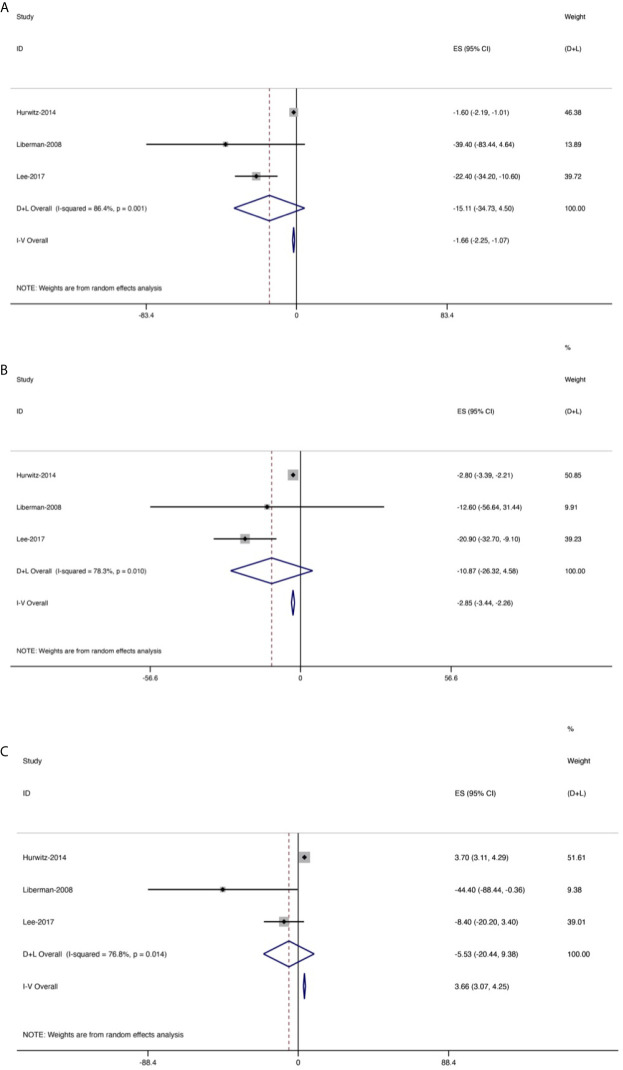
Forest plot for changes from baseline in the oral morphine equivalent dose (OMEDD) at each evaluation point after treatment of patients with bone metastases. **(A)** At 2 weeks. **(B)** At 1 month. **(C)** At 3 months.

**Figure 7:**
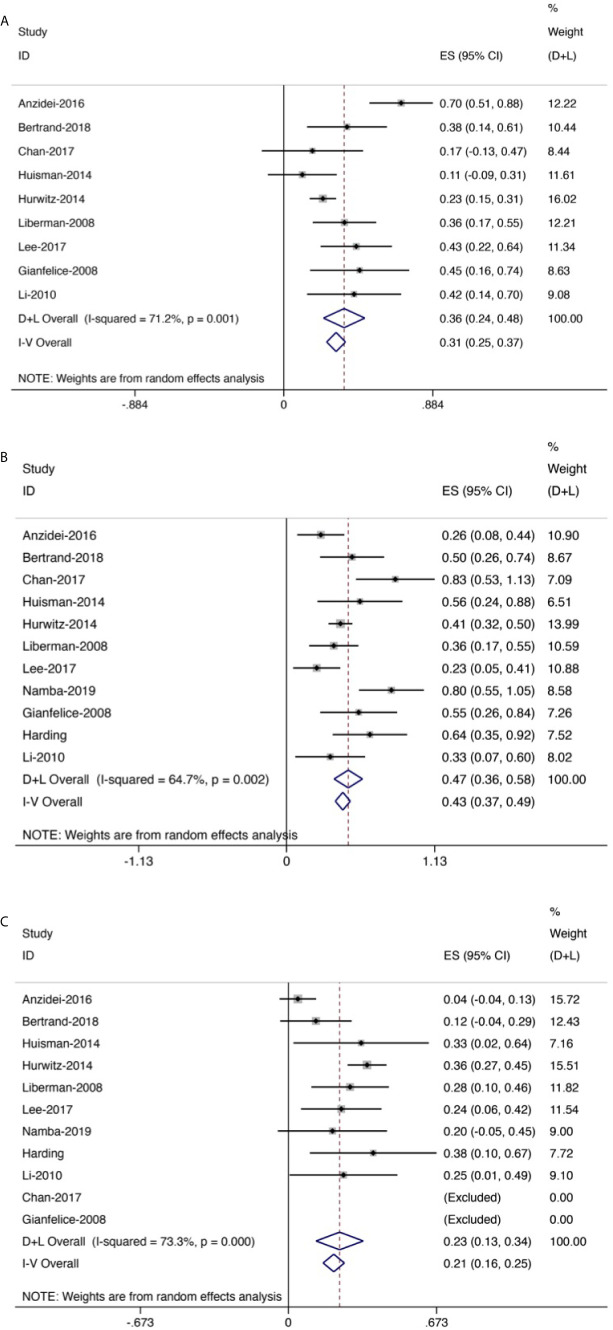
Forest plot for inclusion of all included studies of response rate. **(A)** Complete response rate. **(B)** Partial response rate. **(C)** No response rate.

**Figures 4**–**7** showed the results from meta-analysis of all included studies. The results of pain grade scores are shown in **Figure 4**. Eleven studies (317 patients) assessed pain grade scores at baseline, with a high heterogeneity (*I* ^2^ = 98.1%). The average reported pain scores at baseline was 6.74 (95% CI: 6.30–7.18). Nine studies (268 patients) assessed pain grade scores at 0–1 week with a high heterogeneity (*I* ^2^ = 98.7%). The mean reported pain scores at 0–1 week was 4.15 (95% CI: 3.31–4.99). In addition, 10 trials (291 patients) assessed pain grade scores at 1–5 weeks with a high heterogeneity (*I* ^2^ = 98.2%). The average reported pain scores was 3.09 (95% CI: 2.46–3.72) at 1–5 weeks. Nine trials (289 patients) assessed pain grade scores at 5–14 weeks with a high heterogeneity (*I* ^2^ = 99.7%). The mean reported pain scores was 2.28 (95% CI: 1.37–3.19) at 5–14 weeks.

Generally, the pain scores gradually decreased from baseline to the last follow-up (**Figure 5**). Compared with baseline, the symptom of pain was significantly improved at 0–1 week, with the mean reduced pain scores of 2.54 (95% CI: 1.92–3.16, *p* < 0.01). Besides, compared with baseline, the pain was further improved at 1–5 weeks with a mean reduced pain score of 3.56 (95% CI: 3.11–4.02, *p* < 0.01). Moreover, we also found significant pain improvement at 5–14 weeks, with a mean reduced pain score of 4.22 (95% CI: 3.68–4.76, *p* < 0.01).

OMEDD was also an important index and three trials (158 patients) assessed OMEDD at 2 weeks after treatment (**Figure 6**), with a high heterogeneity (*I* ^2^ = 86.4%). Change from baseline in OMEDD at 2 weeks after treatment was −15.11 (95% CI: −34.73, 4.5). Additionally, four studies (175 patients) evaluated OMEDD at 1 month after treatment, with a high heterogeneity (*I* ^2^ = 78.3%). Change from baseline in OMEDD at 1 month after treatment was −10.87 (95% CI: −26.32, 4.58). Three trials (158 patients) assessed OMEDD at 3 months after treatment, with a high heterogeneity (*I* ^2^ = 76.8%). Change from baseline in OMEDD at 3 months after treatment was −5.53 (95% CI: −20.44, 9.38).

Eleven trials (256 patients) assessed response rate. The results revealed that the overall CR rate was 0.36 (95% CI: 0.24–0.48, **Figure 7A**) and the overall PR rate was 0.47 (95% CI: 0.36–0.58, **Figure 7B**); the overall NR rate was 0.23 (95% CI: 0.13–0.34, **Figure 7C**).

Two studies used the QLQ-BM22 subscale scores to evaluate the survival quality of patients with bone metastases (19, 41). QLQ-BM22 included functional interference, psychosocial aspects, painful site, and pain characteristics. During the first 2 months, the scores of both studies decreased significantly. As time went by, the scores gradually increased. In addition, two studies used QLQ-C15 (19, 41), one study used QLQ-C30 (40), one study used biomarker evaluation including alkaline phosphatase and lactic acid dehydrogenase (22), and one study used median overall survival time (13). All abovementioned indicators showed good therapeutic effects with the treatment of MRgFUS.

There are 14 studies including 352 patients documenting the complications after MRgFUS. Among these studies, 93 (26.4%) patients had minor complications. The main reported minor complications of MRgFUS included sonication pain, position pain, early postprocedural pain, grade I skin burn, and limbs numbness (**Table 3**). Five (1.42%) patients had major complications, which are composed of fractures, third-degree skin burn, hip flexor neuropathy, and sciatic nerve injury.

**Table 3:** Complications of MRgFUS.

Author-publication time	Number of minor complications	Type of minor complications	Number of major complications	Type of major complications
Anzidei-2016 (27)	0		0	
Bertrand-2018 (32)	0		0	
Catane-2007 (33)	0		0	
Chan-2017 (34)	0		0	
Chen-2018 (19)	0		0	
Huisman-2014 (35)	2	Pain after treatment (*n* = 1), grade I skin burn (*n* = 1)	0	
Hurwitz-2014 (36)	59	Sonication pain (*n* = 36), position pain (*n* = 9) postprocedural pain (*n* = 5), fatigue (*n* = 2) neuropathy (*n* = 1), skin burn (*n* = 1), blood in urine (*n* = 1), fever (*n* = 1), myositis (*n* = 1), skin rash (*n* = 1), skin numbness (*n* = 1)	4	Fracture (*n* = 2), grade III skin burn (*n* = 1), hip flexor neuropathy (*n* = 1)
Li-2010 (22)	15	Grade I skin burns (*n* = 12), grade II skin burn (*n* = 2), affected limbs numbness (*n* = 3)	0	
Liberman-2008 (37)	1	Grade II skin burn (*n* = 1)	1	Sciatic nerve injury (*n* = 1)
Lee-2017 (13)	12	Grade II myositis (*n* = 1), Positioning pain (*n* = 3), Sonication pain (*n* = 7), Dermatitis (*n* = 1)	0	
Gianfelice-2008 (39)	0		0	
Wang-2019 (40)	0		0	
Harding-2018 (41)	0		0	
Gu-2015 (42)	4	Lower limbs numbness (*n* = 1), Sonication pain (*n* = 3)	0	

## Discussion

Pain is the most common symptom of patients with cancer, and more than 70% of patients with bone metastasis experience severe persistent bone pain (43, 44). Generally, radiotherapy, chemotherapy, medication, and surgical treatment can be used to relieve bone pain. However, they are not ideal for the analgesic effects. MRgFUS, a safe, effective, and non-invasive ablation method, is approved for painful metastatic bone tumors by the US Food and Drug Administration (FDA) in 2012. However, its therapeutic effects are not very clear. As the meta-analysis on the efficacy of MRgFUS in alleviating the pain in patients with bone metastases, our results revealed that pain grade scores and the usage amount of OMEDD gradually decreased. The conclusion is consistent with the study reported by Baal et al. (45). Besides, the overall CR rate of MRgFUS was 0.36 (95% CI: 0.24–0.48), with a PR rate of 0.47 (95% CI: 0.36–0.58) and a NR rate of 0.23 (95% CI: 0.13–0.34). In addition, among 352 patients who have undergone MRgFUS, 93 (26.4%) patients had minor complications and 5 (1.42%) patients had major complications.

Most patients who suffered from metastatic bone pain would experience intermittent dull pain initially. Gradually, pain became severe and persistent over a few weeks or months (43, 46). Metastatic bone pain has a complex etiology, such as inflammatory pain and pathological neuralgia. Cancer cells and their related stromal cells may release factors. The released factors can not only promote the pathological growth of nerve fibers and the formation of neuroma (47–49), but also sensitize and activate bone nociceptors, thus attaining peripheral and central nerve hypersensitivity (47, 50), consequently leading to the development and persistence of bone pain.

Therapeutically, conventional RT is the initial option for painful bone metastasis and offers a 60% to 80% response rate (13, 51). However, among these responders, relapses frequently occur with an incidence of 30% (52). MRgFUS is more efficient than RT in terms of long-term pain palliation and high response rate (range from 66% to 87%). Thus, it provides an alternative treatment method to overcome radioresistance and is recommended for patients with bone metastasis for whom RT is considered to have failed (12, 13, 36, 47).

OMEDD is another index to evaluate the effects of pain relief. Morphine is commonly used in bone metastases due to its powerful analgesic effects. Generally, it produces pharmacological effects by simulating the endogenous anti-pain substance enkephalin and activating central nervous opioid receptors. Thus, it works better on persistent dull pain than intermittent sharp pain and visceral colic (53). However, it is prone to generate drug resistance and addiction. As the dose increases, severe conditions such as respiratory depression and even coma death may occur (54). Therefore, the application of morphine should be tightly controlled. With the treatment of MRgFUS, we found that the change from baseline in OMEDD at 2 weeks was −15.11 (95% CI: −34.73, 4.5), indicating that MRgFUS may reduce the use of morphine. Baal et al. used different analysis methods, showing that the average of 55.8% and 33.0% of patients could discontinue or reduce pain medication use after treatment of MRgFUS (45), similar to the conclusion we got.

As MRgFUS realizes real-time magnetic resonance dynamic imaging and dynamic temperature monitoring throughout the operation, its complications are relatively low (12, 55). The summary was consistent with the studies of Gennaro et al. (15) and Baal et al. (45). Nevertheless, it is important to take measures to avoid them. Among all the complications, the most common one was a minor complication—sonication pain, which can be expected and usually goes away 1 day after treatment (28). Additionally, sonication pain can also be reduced by local, regional, or general anesthesia (12). As a major complication, skin burn frequently occurs during the procedure. To reduce its risk, the long-term low-output power MRgFUS or short-term high-output power MRgFUS with skin hypothermy treatment through irradiation intervals is recommended (56).

This study has limitations: First, the clinical heterogeneity was found among different studies, which may be associated with differences in gender, age, location, etc. Second, the included studies in this meta-analysis are single-arm ones and lack randomized controlled trials. Third, the pain score functions as one of the evaluation indicators, but it is subjective and uncertain. Other evaluation indicators, such as biomarker evaluation, are only reported in a few literatures, which make the results insufficient.

In summary, this meta-analysis identifies MRgFUS as a reliable therapeutic option to relieve cancer pain for patients with metastatic bone tumors with controllable related complications. In order to further confirm the effectiveness and safety of MRgFUS in relieving pain in patients with metastatic bone tumors, more detailed, multi-regional, multi-ethnic randomized controlled trials are needed in the future.

## Data Availability Statement

The original contributions presented in the study are included in the article/**Supplementary Material**. Further inquiries can be directed to the corresponding authors.

## Author Contributions

XH screened literature and extracted the data from each included study, analyzed data and wrote the article. RH screened literature and extracted the data from each included study, checked the data processing results. TM carried out data inspection and article revision. HY proposed amendments. DS put forward ideas for articles and made a decision. All authors contributed to the article and approved the submitted version.

## Funding

This study was partially supported by Shanghai Rising-Star Program (No. 21QA1407500), the Shanghai Jiaotong University Medical-Industrial Intersection Project (Key) (ZH2018ZDA18), the National Natural Science Foundation of China (No. 81772856), and the Youth Fund of Shanghai Municipal Health Planning Commission (20174Y0117).

## Conflict of Interest

The authors declare that the research was conducted in the absence of any commercial or financial relationships that could be construed as a potential conflict of interest.

## Publisher’s Note

All claims expressed in this article are solely those of the authors and do not necessarily represent those of their affiliated organizations, or those of the publisher, the editors and the reviewers. Any product that may be evaluated in this article, or claim that may be made by its manufacturer, is not guaranteed or endorsed by the publisher.

## Supplementary Material

Supplementary Figure 1 Funnel plot of pain improvement at different follow-up period compared with baseline.
