# Supplementary figures and images for: The Roles of Magnetic Resonance-Guided Focused Ultrasound in Pain Relief in Patients With Bone Metastases: A Systemic Review and Meta-Analysis
Source: Front Oncol. 2021 Aug 11;11:617295. doi: 10.3389/fonc.2021.617295 (PMC8387143; doi:10.3389/fonc.2021.617295)

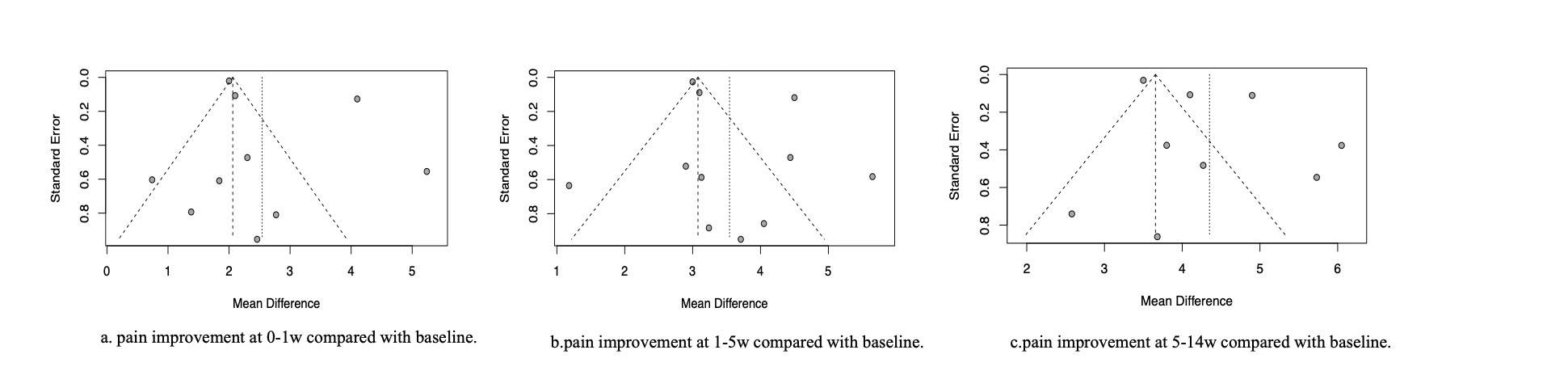

Supplement: Supplementary Figure 1 — Funnel plot of pain improvement at different follow-up period compared with baseline. [file Image_1.jpeg]
